# Bis(μ-9-anthracenemethano­lato)bis­[dimethyl­aluminium(III)]

**DOI:** 10.1107/S1600536809040161

**Published:** 2009-10-07

**Authors:** Chen-Yu Li, Chia-Her Lin, Yi-Chang Liu, Bao-Tsan Ko

**Affiliations:** a Department of Chemistry, Chung Yuan Christian University, Chung-Li 320, Taiwan

## Abstract

The title complex, [Al_2_(CH_3_)_4_(C_15_H_11_O)_2_], is dimeric bridged through the O atoms of the 9-anthracenemethano­late anions. Each Al atom is tetra­coordinated by two bridging O atoms from two different 9-anthracenemethano­late ligands and by two C atoms from two methyl groups, forming a distorted tetra­hedral environment. The average Al—O bond distance in the Al_2_O_2_ core is 1.845 Å.

## Related literature

For background to metal complex-catalysed ring-opening polymerization of lactones/lactides, see: Liu *et al.* (2001 ▶); Wu *et al.* (2006 ▶). For related structures, see: Lin *et al.* (1999 ▶); Lou *et al.* (2002 ▶).
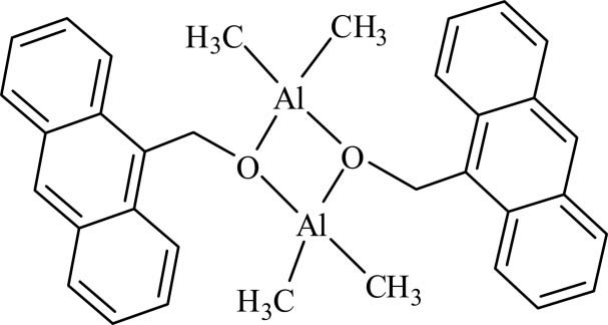


## Experimental

### 

#### Crystal data

[Al_2_(CH_3_)_4_(C_15_H_11_O)_2_]
*M* *_r_* = 528.57
Triclinic, 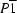

*a* = 7.7852 (3) Å
*b* = 11.3804 (4) Å
*c* = 17.6749 (6) Å
α = 85.683 (2)°
β = 79.883 (2)°
γ = 74.617 (2)°
*V* = 1485.72 (9) Å^3^
*Z* = 2
Mo *K*α radiation
μ = 0.13 mm^−1^
*T* = 296 K
0.45 × 0.38 × 0.32 mm

#### Data collection

Bruker APEXII CCD diffractometer
Absorption correction: multi-scan (*SADABS*; Bruker, 2008 ▶) *T*_min_ = 0.945, *T*_max_ = 0.960
31484 measured reflections
7298 independent reflections
4944 reflections with *I* > 2σ(*I*)
*R*_int_ = 0.033

#### Refinement

*R*[*F* ^2^ > 2σ(*F* ^2^)] = 0.058
*wR*(*F* ^2^) = 0.234
*S* = 1.01
7298 reflections
343 parameters
H-atom parameters constrained
Δρ_max_ = 0.35 e Å^−3^
Δρ_min_ = −0.27 e Å^−3^

### 

Data collection: *APEX2* (Bruker, 2008 ▶); cell refinement: *SAINT-Plus* (Bruker, 2008 ▶); data reduction: *SAINT-Plus*; program(s) used to solve structure: *SHELXS97* (Sheldrick, 2008 ▶); program(s) used to refine structure: *SHELXL97* (Sheldrick, 2008 ▶); molecular graphics: *SHELXTL* (Sheldrick, 2008 ▶); software used to prepare material for publication: *SHELXTL*.

## Supplementary Material

Crystal structure: contains datablocks I, global. DOI: 10.1107/S1600536809040161/rk2172sup1.cif

Structure factors: contains datablocks I. DOI: 10.1107/S1600536809040161/rk2172Isup2.hkl

Additional supplementary materials:  crystallographic information; 3D view; checkCIF report

## Figures and Tables

**Fig. 1.**
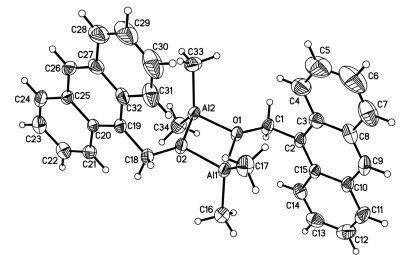
A view of the molecular structure of I with the atom numbering scheme. Displacement ellipsoids are drawn at the 30% probability level. H atoms are presented as a small spheres of arbitrary radius.

**Fig. 2.**
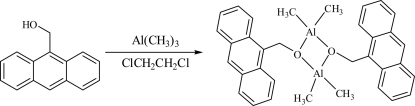
The title compound, I, (reaction path scheme).

## supplementary crystallographic information

### Comment

During the last decade, it has been of great interest to develop new catalytic/initiating systems for the preparation of aliphatic polyesters, such as *poly*(ε-caprolactone) and *poly*(lactide). Metal complex-catalyzed ring-opening polymerization (*ROP*) of lactones/lactides has been proven to be the most promising method to synthesize these polymers (Wu *et al.*, 2006). Among them, a variety of main group metal complexes, such as magnesium, zinc and lithium as well as aluminium complexes have been reported to be efficient initiators/catalysts. In particular, Liu *et al.* (2001) have reported the aluminium benzylalkoxide complexes supported by the bulky bisphenolate ligand and these complexes have been demonstrated as efficient initiators to catalyze *ROP* of cyclic esters. Recently, our group is interested in the synthesis and preparation of aluminium complexes derived from the 9-anthracenemethanolate ligands. The compound, 9-anthracenemethanol has been proven as a useful initiator to initiate living cationic polymerization of δ-valerolactone in the presence of HCl^.^*Et*_2_O (Lou *et al.*, 2002). We report herein the synthesis and crystal structure of the 9-anthracenemethanolate ligand incorporated Al^III^ complex, I, a potential initiator for the *ROP* of ε-caprolactone (Fig. 2).

The solid structure of I reveals a dimeric Al^III^ complex (Fig. 1), doubly bridged through the O atoms of the 9-anthracenemethanolate anions. The geometry around each Al atom is four-coordinated with a distorted tetrahedral environment in which two bridging O atoms come from two different 9-anthracenemethanolate ligands and two C atoms are from two methyl groups. The average bond distance of Al-O in the Al_2_O_2_ core of 1.8453 (14)Å is within a normal range for an Al_2_O_2_ ring of four-coordinated aluminium complexes (Lin *et al.*, 1999).

### Experimental

The title compound I was synthesized by the following procedures (Fig. 2): to a rapidly stirred solution of 9-anthracenemethanol (0.21 g, 1.0 mmol) in 1,2-dichloroethane (20 ml) was slowly added Al*Me*_3_ (0.6 ml, 2.0 *M* in toluene, 1.2 mmol). The mixture was further stirred at room temperature for 4 h and then dried under vacuum. The residue was extracted with 1,2-dichloroethane (10 ml), and the saturated solution was cooled to 273 K, yielding colourless crystals. Yield: 0.22 g (83%). ^1^H NMR (CDCl_3_, p.p.m.): δ 7.43-8.47 (18*H*, m, *Ar*H), 5.67 (4*H*, s, CH_2_), -1.39 (12*H*, s, AlCH_3_).

### Refinement

The H atoms were placed in idealized positions and constrained to ride on their parent atoms, with C–H = 0.93 Å with *U*_iso_(H) = 1.2 *U*_eq_(C) for phenyl hydrogen; 0.96 Å with *U*_iso_(H) = 1.5 *U*_eq_(C) for CH_3_ group; 0.97 Å with *U*_iso_(H) = 1.2 *U*_eq_(C) for CH_2_ group.

### Crystal data

**Table:**

[Al_2_(CH_3_)_4_(C_15_H_11_O)_2_]	*Z* = 2
*M**_r_* = 528.57	*F*(000) = 560
Triclinic, *P*1	*D*_x_ = 1.181 Mg m^−^^3^
Hall symbol: -P 1	Mo *K*α radiation, λ = 0.71073 Å
*a* = 7.7852 (3) Å	Cell parameters from 9970 reflections
*b* = 11.3804 (4) Å	θ = 2.2–28.2°
*c* = 17.6749 (6) Å	µ = 0.13 mm^−^^1^
α = 85.683 (2)°	*T* = 296 K
β = 79.883 (2)°	Block, colourless
γ = 74.617 (2)°	0.45 × 0.38 × 0.32 mm
*V* = 1485.72 (9) Å^3^	

### Data collection

**Table:**

Bruker APEXII CCD diffractometer	7298 independent reflections
Radiation source: fine-focus sealed tube	4944 reflections with *I* > 2σ(*I*)
graphite	*R*_int_ = 0.033
Detector resolution: 8.3333 pixels mm^-1^	θ_max_ = 28.4°, θ_min_ = 1.9°
φ and ω scans	*h* = −10→10
Absorption correction: multi-scan (*SADABS*; Bruker, 2008)	*k* = −14→15
*T*_min_ = 0.945, *T*_max_ = 0.960	*l* = −23→23
31484 measured reflections	

### Refinement

**Table:**

Refinement on *F*^2^	Primary atom site location: structure-invariant direct methods
Least-squares matrix: full	Secondary atom site location: difference Fourier map
*R*[*F*^2^ > 2σ(*F*^2^)] = 0.058	Hydrogen site location: inferred from neighbouring sites
*wR*(*F*^2^) = 0.234	H-atom parameters constrained
*S* = 1.01	*w* = 1/[σ^2^(*F*_o_^2^) + (0.17*P*)^2^] where *P* = (*F*_o_^2^ + 2*F*_c_^2^)/3
7298 reflections	(Δ/σ)_max_ = 0.002
343 parameters	Δρ_max_ = 0.35 e Å^−^^3^
0 restraints	Δρ_min_ = −0.27 e Å^−^^3^

### Special details

**Table:**

Geometry. All s.u.'s (except the s.u. in the dihedral angle between two l.s. planes) are estimated using the full covariance matrix. The cell s.u.'s are taken into account individually in the estimation of s.u.'s in distances, angles and torsion angles; correlations between s.u.'s in cell parameters are only used when they are defined by crystal symmetry. An approximate (isotropic) treatment of cell s.u.'s is used for estimating s.u.'s involving l.s. planes.
Refinement. Refinement of *F*^2^ against ALL reflections. The weighted *R*-factor *wR* and goodness of fit *S* are based on *F*^2^, conventional *R*-factors *R* are based on *F*, with *F* set to zero for negative *F*^2^. The threshold expression of *F*^2^ > σ(*F*^2^) is used only for calculating *R*-factors(gt) *etc*. and is not relevant to the choice of reflections for refinement. *R*-factors based on *F*^2^ are statistically about twice as large as those based on *F*, and *R*-factors based on ALL data will be even larger.

### Fractional atomic coordinates and isotropic or equivalent isotropic displacement parameters (Å^2^)

**Table:**

	*x*	*y*	*z*	*U*_iso_*/*U*_eq_	
Al1	0.71289 (7)	0.62997 (5)	0.30278 (4)	0.0483 (2)	
Al2	0.36490 (7)	0.76614 (5)	0.27971 (3)	0.0449 (2)	
O1	0.50638 (17)	0.60755 (11)	0.27575 (8)	0.0466 (3)	
O2	0.57693 (17)	0.79058 (12)	0.29925 (9)	0.0501 (4)	
C1	0.4531 (3)	0.50037 (18)	0.26509 (14)	0.0550 (5)	
H1A	0.3752	0.5170	0.2262	0.066*	
H1B	0.3847	0.4779	0.3128	0.066*	
C2	0.6143 (2)	0.39518 (17)	0.24061 (12)	0.0460 (4)	
C3	0.6999 (3)	0.3874 (2)	0.16343 (13)	0.0571 (5)	
C4	0.6426 (5)	0.4791 (3)	0.10646 (17)	0.0878 (9)	
H4A	0.5446	0.5454	0.1197	0.105*	
C5	0.7327 (7)	0.4689 (5)	0.0324 (2)	0.1269 (16)	
H5A	0.6961	0.5290	−0.0043	0.152*	
C6	0.8791 (8)	0.3689 (5)	0.0114 (2)	0.143 (2)	
H6A	0.9359	0.3626	−0.0396	0.171*	
C7	0.9404 (5)	0.2806 (4)	0.06366 (19)	0.1063 (12)	
H7A	1.0406	0.2165	0.0487	0.128*	
C8	0.8509 (3)	0.2866 (2)	0.14130 (14)	0.0662 (7)	
C9	0.9068 (3)	0.1975 (2)	0.19499 (16)	0.0651 (6)	
H9A	1.0028	0.1310	0.1794	0.078*	
C10	0.8260 (3)	0.20243 (18)	0.27155 (13)	0.0509 (5)	
C11	0.8872 (3)	0.1102 (2)	0.32601 (19)	0.0709 (7)	
H11A	0.9810	0.0429	0.3098	0.085*	
C12	0.8137 (4)	0.1174 (3)	0.40009 (19)	0.0814 (8)	
H12A	0.8572	0.0564	0.4351	0.098*	
C13	0.6709 (4)	0.2172 (3)	0.42484 (15)	0.0720 (7)	
H13A	0.6209	0.2218	0.4767	0.086*	
C14	0.6036 (3)	0.3072 (2)	0.37536 (13)	0.0580 (6)	
H14A	0.5074	0.3718	0.3938	0.070*	
C15	0.6778 (2)	0.30507 (17)	0.29495 (11)	0.0435 (4)	
C16	0.7421 (4)	0.5780 (2)	0.40803 (15)	0.0778 (8)	
H16A	0.8646	0.5327	0.4093	0.117*	
H16B	0.6624	0.5273	0.4276	0.117*	
H16C	0.7136	0.6482	0.4392	0.117*	
C17	0.9212 (3)	0.6023 (2)	0.22087 (17)	0.0745 (7)	
H17A	1.0257	0.5541	0.2408	0.112*	
H17B	0.9420	0.6792	0.2013	0.112*	
H17C	0.8986	0.5600	0.1802	0.112*	
C18	0.6307 (3)	0.89486 (18)	0.31628 (13)	0.0550 (5)	
H18A	0.7572	0.8851	0.2951	0.066*	
H18B	0.6175	0.9003	0.3716	0.066*	
C19	0.5213 (3)	1.01085 (17)	0.28411 (11)	0.0450 (4)	
C20	0.3866 (2)	1.09329 (17)	0.33176 (10)	0.0401 (4)	
C21	0.3403 (3)	1.0730 (2)	0.41330 (12)	0.0514 (5)	
H21A	0.3966	1.0001	0.4364	0.062*	
C22	0.2155 (3)	1.1591 (2)	0.45698 (14)	0.0625 (6)	
H22A	0.1897	1.1442	0.5097	0.075*	
C23	0.1249 (3)	1.2691 (2)	0.42521 (15)	0.0659 (6)	
H23A	0.0414	1.3271	0.4565	0.079*	
C24	0.1592 (3)	1.2906 (2)	0.34909 (15)	0.0601 (6)	
H24A	0.0965	1.3632	0.3279	0.072*	
C25	0.2891 (3)	1.20518 (18)	0.30016 (12)	0.0460 (4)	
C26	0.3239 (3)	1.2290 (2)	0.22149 (13)	0.0574 (6)	
H26A	0.2579	1.3008	0.2007	0.069*	
C27	0.4547 (4)	1.1484 (2)	0.17324 (13)	0.0614 (6)	
C28	0.4924 (5)	1.1736 (3)	0.09214 (15)	0.0921 (10)	
H28A	0.4240	1.2435	0.0705	0.110*	
C29	0.6261 (7)	1.0967 (4)	0.04722 (17)	0.1195 (15)	
H29A	0.6502	1.1150	−0.0051	0.143*	
C30	0.7283 (6)	0.9909 (4)	0.0774 (2)	0.1214 (15)	
H30A	0.8206	0.9396	0.0452	0.146*	
C31	0.6957 (4)	0.9609 (3)	0.15368 (16)	0.0860 (9)	
H31A	0.7643	0.8886	0.1728	0.103*	
C32	0.5583 (3)	1.0386 (2)	0.20398 (12)	0.0560 (5)	
C33	0.3002 (4)	0.8271 (3)	0.17994 (14)	0.0738 (7)	
H33A	0.1750	0.8706	0.1863	0.111*	
H33B	0.3209	0.7599	0.1469	0.111*	
H33C	0.3728	0.8810	0.1574	0.111*	
C34	0.1772 (3)	0.7840 (2)	0.37059 (14)	0.0673 (6)	
H34A	0.0645	0.8314	0.3570	0.101*	
H34B	0.2097	0.8245	0.4095	0.101*	
H34C	0.1652	0.7049	0.3898	0.101*	

### Atomic displacement parameters (Å^2^)

**Table:**

	*U*^11^	*U*^22^	*U*^33^	*U*^12^	*U*^13^	*U*^23^
Al1	0.0401 (3)	0.0352 (3)	0.0677 (4)	−0.0034 (2)	−0.0115 (3)	−0.0059 (3)
Al2	0.0410 (3)	0.0354 (3)	0.0551 (4)	−0.0032 (2)	−0.0095 (2)	−0.0001 (2)
O1	0.0405 (7)	0.0330 (7)	0.0656 (8)	−0.0061 (5)	−0.0100 (6)	−0.0060 (6)
O2	0.0437 (7)	0.0330 (7)	0.0742 (9)	−0.0088 (5)	−0.0121 (6)	−0.0041 (6)
C1	0.0448 (10)	0.0343 (11)	0.0875 (15)	−0.0110 (8)	−0.0136 (10)	−0.0016 (10)
C2	0.0431 (9)	0.0333 (10)	0.0643 (12)	−0.0127 (7)	−0.0097 (8)	−0.0056 (8)
C3	0.0709 (14)	0.0490 (12)	0.0601 (12)	−0.0285 (10)	−0.0148 (10)	0.0017 (10)
C4	0.124 (3)	0.080 (2)	0.0741 (17)	−0.0458 (18)	−0.0311 (16)	0.0150 (14)
C5	0.195 (5)	0.136 (4)	0.071 (2)	−0.085 (4)	−0.028 (3)	0.032 (2)
C6	0.201 (5)	0.191 (6)	0.059 (2)	−0.112 (5)	0.021 (2)	−0.015 (3)
C7	0.121 (3)	0.120 (3)	0.077 (2)	−0.050 (2)	0.0299 (19)	−0.036 (2)
C8	0.0713 (15)	0.0648 (16)	0.0659 (14)	−0.0290 (12)	0.0059 (11)	−0.0211 (12)
C9	0.0531 (12)	0.0468 (13)	0.0918 (17)	−0.0092 (10)	0.0008 (11)	−0.0232 (12)
C10	0.0446 (10)	0.0341 (10)	0.0768 (14)	−0.0114 (8)	−0.0125 (9)	−0.0082 (9)
C11	0.0621 (14)	0.0389 (13)	0.119 (2)	−0.0142 (10)	−0.0352 (14)	0.0051 (13)
C12	0.092 (2)	0.0694 (19)	0.102 (2)	−0.0431 (16)	−0.0486 (17)	0.0321 (16)
C13	0.0897 (18)	0.082 (2)	0.0616 (14)	−0.0488 (16)	−0.0242 (13)	0.0115 (13)
C14	0.0592 (12)	0.0580 (14)	0.0635 (13)	−0.0270 (10)	−0.0065 (10)	−0.0096 (10)
C15	0.0408 (9)	0.0344 (10)	0.0598 (11)	−0.0147 (7)	−0.0107 (8)	−0.0057 (8)
C16	0.0953 (19)	0.0590 (16)	0.0813 (17)	−0.0070 (14)	−0.0378 (15)	−0.0061 (13)
C17	0.0463 (12)	0.0707 (18)	0.1021 (19)	−0.0134 (11)	0.0024 (12)	−0.0149 (14)
C18	0.0538 (11)	0.0385 (11)	0.0758 (14)	−0.0087 (9)	−0.0203 (10)	−0.0096 (10)
C19	0.0500 (10)	0.0348 (10)	0.0559 (11)	−0.0174 (8)	−0.0141 (8)	−0.0006 (8)
C20	0.0446 (9)	0.0337 (9)	0.0489 (10)	−0.0186 (7)	−0.0141 (7)	0.0017 (7)
C21	0.0567 (11)	0.0488 (12)	0.0544 (11)	−0.0226 (9)	−0.0135 (9)	0.0064 (9)
C22	0.0649 (13)	0.0680 (16)	0.0582 (12)	−0.0289 (12)	0.0008 (10)	−0.0070 (11)
C23	0.0548 (13)	0.0591 (15)	0.0837 (17)	−0.0167 (11)	−0.0018 (11)	−0.0162 (12)
C24	0.0520 (12)	0.0388 (12)	0.0912 (17)	−0.0103 (9)	−0.0188 (11)	−0.0012 (11)
C25	0.0467 (10)	0.0377 (10)	0.0603 (11)	−0.0193 (8)	−0.0165 (8)	0.0055 (8)
C26	0.0707 (14)	0.0456 (12)	0.0670 (13)	−0.0274 (10)	−0.0292 (11)	0.0166 (10)
C27	0.0900 (17)	0.0613 (15)	0.0494 (11)	−0.0433 (13)	−0.0216 (11)	0.0061 (10)
C28	0.144 (3)	0.096 (2)	0.0536 (14)	−0.060 (2)	−0.0243 (16)	0.0117 (15)
C29	0.201 (4)	0.131 (4)	0.0437 (14)	−0.086 (3)	0.003 (2)	−0.0071 (18)
C30	0.177 (4)	0.118 (3)	0.073 (2)	−0.070 (3)	0.036 (2)	−0.038 (2)
C31	0.106 (2)	0.0741 (19)	0.0757 (17)	−0.0322 (16)	0.0162 (15)	−0.0268 (14)
C32	0.0733 (14)	0.0493 (13)	0.0544 (11)	−0.0314 (11)	−0.0076 (10)	−0.0090 (9)
C33	0.0879 (18)	0.0706 (18)	0.0672 (15)	−0.0211 (14)	−0.0297 (13)	0.0142 (12)
C34	0.0529 (12)	0.0675 (16)	0.0729 (15)	−0.0059 (11)	−0.0001 (10)	−0.0059 (12)

### Geometric parameters (Å, °)

**Table:**

Al1—O1	1.8396 (13)	C16—H16B	0.9600
Al1—O2	1.8560 (14)	C16—H16C	0.9600
Al1—C16	1.943 (3)	C17—H17A	0.9600
Al1—C17	1.949 (2)	C17—H17B	0.9600
Al1—Al2	2.8236 (8)	C17—H17C	0.9600
Al2—O2	1.8384 (14)	C18—C19	1.501 (3)
Al2—O1	1.8473 (14)	C18—H18A	0.9700
Al2—C33	1.946 (2)	C18—H18B	0.9700
Al2—C34	1.956 (2)	C19—C20	1.406 (3)
O1—C1	1.424 (2)	C19—C32	1.424 (3)
O2—C18	1.427 (2)	C20—C25	1.428 (3)
C1—C2	1.512 (3)	C20—C21	1.439 (3)
C1—H1A	0.9700	C21—C22	1.359 (3)
C1—H1B	0.9700	C21—H21A	0.9300
C2—C15	1.403 (3)	C22—C23	1.395 (4)
C2—C3	1.407 (3)	C22—H22A	0.9300
C3—C8	1.429 (3)	C23—C24	1.342 (3)
C3—C4	1.433 (4)	C23—H23A	0.9300
C4—C5	1.369 (5)	C24—C25	1.419 (3)
C4—H4A	0.9300	C24—H24A	0.9300
C5—C6	1.400 (6)	C25—C26	1.389 (3)
C5—H5A	0.9300	C26—C27	1.387 (4)
C6—C7	1.360 (6)	C26—H26A	0.9300
C6—H6A	0.9300	C27—C32	1.422 (4)
C7—C8	1.423 (4)	C27—C28	1.435 (3)
C7—H7A	0.9300	C28—C29	1.345 (5)
C8—C9	1.373 (4)	C28—H28A	0.9300
C9—C10	1.387 (3)	C29—C30	1.382 (6)
C9—H9A	0.9300	C29—H29A	0.9300
C10—C11	1.414 (3)	C30—C31	1.362 (4)
C10—C15	1.434 (3)	C30—H30A	0.9300
C11—C12	1.333 (4)	C31—C32	1.414 (3)
C11—H11A	0.9300	C31—H31A	0.9300
C12—C13	1.396 (4)	C33—H33A	0.9600
C12—H12A	0.9300	C33—H33B	0.9600
C13—C14	1.353 (4)	C33—H33C	0.9600
C13—H13A	0.9300	C34—H34A	0.9600
C14—C15	1.437 (3)	C34—H34B	0.9600
C14—H14A	0.9300	C34—H34C	0.9600
C16—H16A	0.9600		
			
O1—Al1—O2	79.89 (6)	C10—C15—C14	115.7 (2)
O1—Al1—C16	113.50 (10)	Al1—C16—H16A	109.5
O2—Al1—C16	110.32 (9)	Al1—C16—H16B	109.5
O1—Al1—C17	114.72 (10)	H16A—C16—H16B	109.5
O2—Al1—C17	110.62 (10)	Al1—C16—H16C	109.5
C16—Al1—C17	120.46 (13)	H16A—C16—H16C	109.5
O1—Al1—Al2	40.12 (4)	H16B—C16—H16C	109.5
O2—Al1—Al2	39.93 (4)	Al1—C17—H17A	109.5
C16—Al1—Al2	116.40 (9)	Al1—C17—H17B	109.5
C17—Al1—Al2	122.85 (9)	H17A—C17—H17B	109.5
O2—Al2—O1	80.15 (6)	Al1—C17—H17C	109.5
O2—Al2—C33	115.53 (10)	H17A—C17—H17C	109.5
O1—Al2—C33	111.86 (10)	H17B—C17—H17C	109.5
O2—Al2—C34	112.98 (9)	O2—C18—C19	112.21 (16)
O1—Al2—C34	109.75 (9)	O2—C18—H18A	109.2
C33—Al2—C34	119.67 (12)	C19—C18—H18A	109.2
O2—Al2—Al1	40.39 (4)	O2—C18—H18B	109.2
O1—Al2—Al1	39.92 (4)	C19—C18—H18B	109.2
C33—Al2—Al1	124.36 (9)	H18A—C18—H18B	107.9
C34—Al2—Al1	115.69 (8)	C20—C19—C32	119.69 (19)
C1—O1—Al1	132.01 (11)	C20—C19—C18	121.31 (18)
C1—O1—Al2	127.49 (11)	C32—C19—C18	118.98 (19)
Al1—O1—Al2	99.97 (6)	C19—C20—C25	120.19 (18)
C18—O2—Al2	134.10 (12)	C19—C20—C21	123.53 (19)
C18—O2—Al1	125.88 (11)	C25—C20—C21	116.28 (18)
Al2—O2—Al1	99.69 (6)	C22—C21—C20	120.8 (2)
O1—C1—C2	111.61 (15)	C22—C21—H21A	119.6
O1—C1—H1A	109.3	C20—C21—H21A	119.6
C2—C1—H1A	109.3	C21—C22—C23	122.0 (2)
O1—C1—H1B	109.3	C21—C22—H22A	119.0
C2—C1—H1B	109.3	C23—C22—H22A	119.0
H1A—C1—H1B	108.0	C24—C23—C22	119.4 (2)
C15—C2—C3	120.10 (19)	C24—C23—H23A	120.3
C15—C2—C1	119.84 (19)	C22—C23—H23A	120.3
C3—C2—C1	120.1 (2)	C23—C24—C25	121.5 (2)
C2—C3—C8	119.2 (2)	C23—C24—H24A	119.2
C2—C3—C4	122.0 (2)	C25—C24—H24A	119.2
C8—C3—C4	118.8 (2)	C26—C25—C24	120.9 (2)
C5—C4—C3	119.8 (4)	C26—C25—C20	119.19 (19)
C5—C4—H4A	120.1	C24—C25—C20	119.94 (19)
C3—C4—H4A	120.1	C27—C26—C25	121.6 (2)
C4—C5—C6	120.7 (4)	C27—C26—H26A	119.2
C4—C5—H5A	119.7	C25—C26—H26A	119.2
C6—C5—H5A	119.7	C26—C27—C32	120.1 (2)
C7—C6—C5	121.6 (3)	C26—C27—C28	121.7 (3)
C7—C6—H6A	119.2	C32—C27—C28	118.2 (3)
C5—C6—H6A	119.2	C29—C28—C27	120.4 (3)
C6—C7—C8	119.9 (4)	C29—C28—H28A	119.8
C6—C7—H7A	120.1	C27—C28—H28A	119.8
C8—C7—H7A	120.1	C28—C29—C30	121.2 (3)
C9—C8—C7	121.3 (3)	C28—C29—H29A	119.4
C9—C8—C3	119.6 (2)	C30—C29—H29A	119.4
C7—C8—C3	119.1 (3)	C31—C30—C29	120.9 (3)
C8—C9—C10	122.7 (2)	C31—C30—H30A	119.6
C8—C9—H9A	118.7	C29—C30—H30A	119.6
C10—C9—H9A	118.7	C30—C31—C32	120.6 (3)
C9—C10—C11	121.7 (2)	C30—C31—H31A	119.7
C9—C10—C15	118.2 (2)	C32—C31—H31A	119.7
C11—C10—C15	120.1 (2)	C31—C32—C27	118.7 (2)
C12—C11—C10	121.5 (3)	C31—C32—C19	122.2 (2)
C12—C11—H11A	119.2	C27—C32—C19	119.2 (2)
C10—C11—H11A	119.2	Al2—C33—H33A	109.5
C11—C12—C13	119.7 (2)	Al2—C33—H33B	109.5
C11—C12—H12A	120.2	H33A—C33—H33B	109.5
C13—C12—H12A	120.2	Al2—C33—H33C	109.5
C14—C13—C12	121.7 (3)	H33A—C33—H33C	109.5
C14—C13—H13A	119.1	H33B—C33—H33C	109.5
C12—C13—H13A	119.1	Al2—C34—H34A	109.5
C13—C14—C15	121.3 (2)	Al2—C34—H34B	109.5
C13—C14—H14A	119.4	H34A—C34—H34B	109.5
C15—C14—H14A	119.4	Al2—C34—H34C	109.5
C2—C15—C10	120.14 (19)	H34A—C34—H34C	109.5
C2—C15—C14	124.20 (19)	H34B—C34—H34C	109.5
			
O1—Al1—Al2—O2	−173.54 (10)	C2—C3—C8—C7	−178.3 (2)
C16—Al1—Al2—O2	90.62 (12)	C4—C3—C8—C7	0.3 (3)
C17—Al1—Al2—O2	−83.19 (12)	C7—C8—C9—C10	178.0 (2)
O2—Al1—Al2—O1	173.54 (10)	C3—C8—C9—C10	−2.1 (3)
C16—Al1—Al2—O1	−95.83 (12)	C8—C9—C10—C11	−179.3 (2)
C17—Al1—Al2—O1	90.35 (12)	C8—C9—C10—C15	0.2 (3)
O1—Al1—Al2—C33	−83.44 (12)	C9—C10—C11—C12	177.5 (2)
O2—Al1—Al2—C33	90.10 (12)	C15—C10—C11—C12	−2.0 (3)
C16—Al1—Al2—C33	−179.27 (12)	C10—C11—C12—C13	1.0 (4)
C17—Al1—Al2—C33	6.91 (14)	C11—C12—C13—C14	0.4 (4)
O1—Al1—Al2—C34	90.53 (11)	C12—C13—C14—C15	−0.8 (3)
O2—Al1—Al2—C34	−95.92 (12)	C3—C2—C15—C10	−2.2 (3)
C16—Al1—Al2—C34	−5.30 (13)	C1—C2—C15—C10	177.45 (16)
C17—Al1—Al2—C34	−179.11 (12)	C3—C2—C15—C14	177.77 (17)
O2—Al1—O1—C1	−176.03 (18)	C1—C2—C15—C14	−2.6 (3)
C16—Al1—O1—C1	−68.15 (19)	C9—C10—C15—C2	2.0 (3)
C17—Al1—O1—C1	75.81 (19)	C11—C10—C15—C2	−178.52 (18)
Al2—Al1—O1—C1	−171.8 (2)	C9—C10—C15—C14	−177.99 (17)
O2—Al1—O1—Al2	−4.20 (6)	C11—C10—C15—C14	1.5 (3)
C16—Al1—O1—Al2	103.68 (10)	C13—C14—C15—C2	179.87 (19)
C17—Al1—O1—Al2	−112.36 (10)	C13—C14—C15—C10	−0.2 (3)
O2—Al2—O1—C1	176.59 (17)	Al2—O2—C18—C19	−26.6 (3)
C33—Al2—O1—C1	−69.73 (19)	Al1—O2—C18—C19	161.38 (14)
C34—Al2—O1—C1	65.57 (18)	O2—C18—C19—C20	105.0 (2)
Al1—Al2—O1—C1	172.35 (19)	O2—C18—C19—C32	−76.5 (2)
O2—Al2—O1—Al1	4.24 (6)	C32—C19—C20—C25	−0.9 (3)
C33—Al2—O1—Al1	117.92 (11)	C18—C19—C20—C25	177.62 (16)
C34—Al2—O1—Al1	−106.78 (10)	C32—C19—C20—C21	−179.83 (17)
O1—Al2—O2—C18	−177.66 (19)	C18—C19—C20—C21	−1.3 (3)
C33—Al2—O2—C18	72.7 (2)	C19—C20—C21—C22	176.45 (18)
C34—Al2—O2—C18	−70.3 (2)	C25—C20—C21—C22	−2.5 (3)
Al1—Al2—O2—C18	−173.5 (2)	C20—C21—C22—C23	1.1 (3)
O1—Al2—O2—Al1	−4.20 (6)	C21—C22—C23—C24	1.0 (3)
C33—Al2—O2—Al1	−113.83 (11)	C22—C23—C24—C25	−1.6 (3)
C34—Al2—O2—Al1	103.19 (10)	C23—C24—C25—C26	−179.6 (2)
O1—Al1—O2—C18	178.43 (17)	C23—C24—C25—C20	0.0 (3)
C16—Al1—O2—C18	66.97 (19)	C19—C20—C25—C26	2.6 (3)
C17—Al1—O2—C18	−68.82 (18)	C21—C20—C25—C26	−178.35 (16)
Al2—Al1—O2—C18	174.21 (19)	C19—C20—C25—C24	−177.04 (16)
O1—Al1—O2—Al2	4.22 (6)	C21—C20—C25—C24	2.0 (3)
C16—Al1—O2—Al2	−107.24 (11)	C24—C25—C26—C27	177.8 (2)
C17—Al1—O2—Al2	116.97 (10)	C20—C25—C26—C27	−1.9 (3)
Al1—O1—C1—C2	−27.9 (3)	C25—C26—C27—C32	−0.6 (3)
Al2—O1—C1—C2	162.27 (14)	C25—C26—C27—C28	−179.2 (2)
O1—C1—C2—C15	100.4 (2)	C26—C27—C28—C29	176.7 (3)
O1—C1—C2—C3	−79.9 (2)	C32—C27—C28—C29	−2.0 (4)
C15—C2—C3—C8	0.3 (3)	C27—C28—C29—C30	1.1 (6)
C1—C2—C3—C8	−179.36 (18)	C28—C29—C30—C31	0.5 (6)
C15—C2—C3—C4	−178.3 (2)	C29—C30—C31—C32	−1.2 (5)
C1—C2—C3—C4	2.1 (3)	C30—C31—C32—C27	0.3 (4)
C2—C3—C4—C5	178.5 (3)	C30—C31—C32—C19	−179.4 (3)
C8—C3—C4—C5	0.0 (4)	C26—C27—C32—C31	−177.4 (2)
C3—C4—C5—C6	0.8 (6)	C28—C27—C32—C31	1.3 (3)
C4—C5—C6—C7	−1.9 (7)	C26—C27—C32—C19	2.3 (3)
C5—C6—C7—C8	2.3 (6)	C28—C27—C32—C19	−179.02 (19)
C6—C7—C8—C9	178.5 (3)	C20—C19—C32—C31	178.1 (2)
C6—C7—C8—C3	−1.4 (5)	C18—C19—C32—C31	−0.4 (3)
C2—C3—C8—C9	1.8 (3)	C20—C19—C32—C27	−1.6 (3)
C4—C3—C8—C9	−179.6 (2)	C18—C19—C32—C27	179.90 (18)
